# Effects of sires with different weight gain potentials and varying planes of nutrition on growth of growing-finishing pigs

**DOI:** 10.1186/2055-0391-56-22

**Published:** 2014-10-28

**Authors:** Duck-Min Ha, Dae-Yun Jung, Man Jong Park, Byung-Chul Park, C Young Lee

**Affiliations:** Regional Animal Industry Center, Gyeongnam National University of Science and Technology, Jinju, 660-758 Korea; Sunjin Co., Ltd, 517-3 Doonchon-dong, Kangdong-gu, Seoul, 134-060 Korea

**Keywords:** Sire, Progeny, Weight gain, Plane of nutrition, Growing-finishing pig

## Abstract

The present study was performed to investigate the effects of two groups of sires with ‘medium’ and ‘high’ weight gain potentials (M-sires and H-sires, respectively) on growth of their progenies on varying planes of nutrition during the growing-finishing period. The ADG of the M-sires’ progeny was greater (P < 0.05) than that of the H-sires’ progeny (0.51 vs. 0.47 kg) during a 26- to 29-d early grower phase beginning from 55 d of age, but the opposite was true (0.66 vs. 0.72 kg) during the latter grower phase. Overall grower-phase ADG was greatest on the high plane of nutrition (H plane) followed by the medium (M) and low (L) planes (0.65, 0.61, and 0.51 kg, respectively; P < 0.05) in the M-sires’ progeny, whereas in the H-sires’ progeny, ADG was greater on the H and M planes vs. L plane (0.63, 0.62, and 0.54 kg, respectively). The ADG of pigs on the M or H plane during the grower phase and switched to the H plane thereafter (M-to-H or H-to-H planes) was greater than that of pigs on the L-to-L planes (0.99 vs. 0.78 kg) during the early finisher phase in the M-sires’ progeny (P < 0.01). However, in the H-sires’ progeny, ADG of pigs on the L-to-L planes did not differ from that of pigs on the M-to-M or H-to-M planes (0.94 vs. 0.96 kg). Results suggest that the H-to-H or H-to-M planes and M-to-M or M-to-L planes are optimal for maximal growth of the M- and H-sires’ progenies, respectively.

## Background

The rate of weight gain or lean gain is a most important economic trait in pig production [1–3]. Breeding pigs have thus been selected for greater ADG/lean gain and less backfat thickness for decades in the Western countries, partly because most consumers prefer lean pork, partly because lean gain is much more efficient energetically than fat gain [4, 5]. Given the genetic background, growth of pigs is determined primarily by nutrition as well as environmental stressors including the presence/absence or severity of diseases and heat stress [6–9].

Backfat thickness, a negative indicator of lean gain, which is known to be moderately correlated with the rate of weight gain [5], decreased through the mid-2000s as a result of selection of breeding pigs for greater lean gain in Korea [10]. However, backfat thickness rebounded by approximately 2 mm during the latter 2000s due primarily to an allegation that lean pigs are less resistant to diseases, especially to the post-weaning multi-systemic wasting syndrome (PMWS) prevalent during that period, and also are less preferred by domestic consumers [11]. This resulted in a comparable increase in the backfat thickness of market pigs with a lag time of approximately 2 years [12]. Currently, the average backfat thickness of market pigs in Korea is 21.3 mm with an average liveweight of 114.5 kg [13]. This is much greater than the estimated 17 mm or less at the same liveweight in the USA [14] where the average liveweight of market pigs was 125.4 kg in 2013 [15]. These data suggest that overall growth potential of growing-finishing pigs in Korea is lower than that in the USA. As an initial step to see the influences of genetic growth potential on growth efficiency and thereby suggesting optimal feeding programs for pigs with different growth potentials, effects of sires with a high weight gain potential vs. a medium potential on growth of their progenies during the growing-finishing period were investigated in the present study.

## Methods

### Breeding

Four Duroc boars of JSR Genetics Ltd. (East Yorkshire, UK) origin, which were judged to have a high weight gain potential (H-sires) based on their breeding indices, were purchased from Darby Genetics Inc. (Anseong, Korea) for the present experiment in the fall of 2011. The other four boars with a medium weight gain potential (M-sires), which were suitable as sires under the carcass grading standard of 2011 [16], were selected from boars available in a commercial farm where the present experiment was performed. The H-sires and M-sires were mated to more than 20 Yorkshire × Landrace ‘M-dams’ of JSR origin per sire group by artificial insemination in mid- February, 2012. The progeny used for the present experiment were born on June 7 and received cross-fostering within the litters born to each sire group, iron injection, and other routine cares for suckling pigs during 21 d of lactation.

### Experimental animals and diets

Weanling pigs were pooled by sex in each progeny from the respective group of sires, randomly distributed to the nursery pens (34 animals per pen), and fed the 3-phase nursery diets as previously described [17]. At 55 d of age, three pens of females and three pens of castrated males in each of the two progeny groups were selected and moved to grower pens by the pen unit for the present experiment beginning from August 1, 2012. Each pen in each progeny group × sex combination was placed on a high, medium, or low plane of nutrition during the grower phase (Table 1) for 55 to 57 d, after which the pigs were subjected to one of the two dietary treatments in each progeny group. In the M-sires’ progeny, pigs on the high or medium plane of nutrition during the grower phase were fed the high (H)- and medium (M)-plane finisher diets during the finisher phases 1 and 2 up to marketing, respectively, whereas those on the low (L) plane of nutrition during the grower phase were fed the L-plane finisher diet during both finisher phases 1 and 2 (Table 2). In the progeny of the H-sires, pigs on the H or M plane of nutrition during the grower phase were fed the M- and L-plane finisher diets during the finisher phases 1 and 2, respectively; those on the L plane of nutrition during the grower phase was fed the L-plane finisher diet during both finisher phases 1 and 2.

**Table 1:** **Declared minimum nutritional values of the commercial diets used in the present study (as-fed basis)**

Item	Grower phase 1^1)^			Grower phase 2^2)^			Finisher^3)^		
	H^4)^	M^5)^	L^6)^	H	M	L	H^7)^	M	L
Crude protein, %	18.2	17.5	15.5	16.6	16.0	15.6	13.5	13.8	13.4
Lysine, %	1.1	1.0	0.8	1.0	0.8	0.8	0.7	0.8	0.7
Crude fat, %	6.0	4.0	3.0	5.0	3.0	3.0	3.0	3.0	2.5
DE, Mcal/kg	3.45	3.35	3.30	3.45	3.30	3.30	3.24	3.30	3.25

^1)-3)^Fed from 16–29 kg, 29–48 kg, and 48–114 kg of body weight, respectively, on average.

^4)-6)^High-, medium, and low planes of nutrition, respectively.

^7)^It was informed from the formulator of the experimental diets that actual nutrients densities of the finisher H were higher than those of the finisher M and also comparable to those of the phase 2 grower M.

**Table 2:** **Experimental design: dietary regimens for the pigs born from two groups of sires with medium and high weight gain potentials**

Sire group		Grower				Finisher				
	Plane of nutrition (PN)	Phase 1		Phase 2		PN	Phase 1		Phase 2	
		Diet^1)^	Days	Diet^1)^	Days		Diet^1)^	Days^2)^	Diet^1)^	Days^2)^
Medium weight gain potential	High (H)	H	26	H	29	H	H	28 or 29	M	38 to 42
	Medium (M)	M	27	M	29					
	Low (L)	L	29	L	28	L	L	35 or 36	L	45 or 46
High weight gain potential	H	H	26	H	29	M	M	30 or 31	L	37 or 38
	M	M	27	M	29					
	L	L	29	L	28	L	L	37	L	38

^1)^See Table 1 for the nutritional composition of the diets for the high (H)-, medium (M)-, and low (L)-planes of nutrition during each phase.

^2)^See Tables 4 and 5 for more details.

A total of 120 pigs in 12 pens (10 animals per pen), which had been selected randomly at the outset of the experiment, were weighed at the beginning as well as at the ends of grower phases 1 and 2 and finisher phases 1 and 2. The animals were transported to a local abattoir on the last day of the finisher phase 2 and slaughtered the following day after overnight lairage. Backfat thickness of the carcass was adjusted to a liveweight of 115 kg as described previously [18, 19]. The number of days required to reach the 115-kg liveweight was estimated as follows: age in days at the end of the finisher phase 2 + (115 kg - liveweight of the finisher phase 2 in kg)/ADG during the finisher phase 2.

The experimental protocol conformed to the guidelines of the Institutional Animal Care and Use Committee (IACUC) at Gyeongnam National University of Science and Technology. The animals used in the present study were treated humanely throughout the study and did not receive any prolonged constraint.

### Statistical analysis

All data were analyzed using SAS (SAS Inst. Inc., Cary, NC, USA). Individual animal was the experimental unit in all analyses. Data for the grower phase were analyzed using the GLM procedure as a completely randomize design with a 2 (sire type) × 2 (sex) × 3 (plane of nutrition) factorial arrangement of treatments. Finisher phase results of the two progeny groups born to their respective sire groups were analyzed separately using the ANOVA procedure for estimation of *P*-values for the fixed errors and the their interactions as well as using the GLM procedure for estimation of the least squares means and comparisons between means by the PDIFF option. Effects of the fixed errors and their interactions and differences between two means were considered to be significant when the corresponding *P*-value was less than 0.05.

## Results

### Grower phase

Growth performance of the animals during the grower phase is shown in Table 3. The effect of sex was not significant in any of the variables (P > 0.05), and thus the results on sex within each sire group’s progeny × plane of nutrition as well *P*-values for sex-associated interactions are not shown in this table for brevity. The ADG during the phase 1 was greater in the M-sires’ progeny than in the H-sires’ progeny (0.51 vs. 0.47 kg; SE = 0.01 kg; P < 0.01). Moreover, ADG during the phase 1 was greater (P < 0.01) in animals on the H or M plane of nutrition (H or M group) than in those on the L plane of nutrition (L group; 0.54, 0.51, and 0.42 kg in the H, M, and L groups, respectively; SE = 0.02 kg). In addition, phase 1 ADG was greater in the H and M groups than in the L group in the progeny of the M-sires whereas in the progeny of the H-sires, ADG was greater only in the H group vs. the L group.

**Table 3:** **Growth performance of the pigs born from two groups of sires with high and medium weight gain potentials, respectively, on varying planes of nutrition during the grower phase beginning from August 1 through September 26, 2012**

Item	Medium potential			High potential			SEM	Sex			***P***-value			
	H^1)^	M^2)^	L^3)^	H^1)^	M^2)^	L^3)^		B^4)^	G^4)^	SEM	Sr^4)^	PN^4)^	Sr × PN	S^4)^
Initial wt, kg	15.2	15.8	16.0	15.7	16.4	14.3	0.44	15.5	15.6	0.25	0.63	0.11	0.02	0.81
Days on feed														
Phase (P) 1	26	27	29	26	27	29								
P 2	29	29	28	29	29	28								
Final wt, kg														
P 1	30.7	30.5	27.6	28.7	29.1	27.0	0.8	28.8	29.0	0.5	0.05	<0.01	0.67	0.77
P 2	51.6	49.7	44.0	49.9	50.4	45.1	1.2	48.8	48.1	0.7	0.96	<0.01	0.46	0.48
ADG														
P 1	0.59	0.54	0.40	0.50	0.47	0.44	0.02	0.49	0.49	0.01	<0.01	<0.01	<0.01	0.87
P 2	0.72	0.66	0.59	0.76	0.77	0.64	0.02	0.71	0.67	0.01	<0.01	<0.01	0.27	0.08
Overall	0.66	0.61	0.49	0.63	0.62	0.54	0.02	0.60	0.58	0.01	0.48	<0.01	0.13	0.26

^1), 2), 3)^H, high plane of nutrition; M, medium plane of nutrition; L, low plane of nutrition. Data are least squares means of 20 animals. Average ADFI for both H, M, and L groups were 1.07, 1.06, and 0.84 kg and 1.78, 1.72, and 2.02 kg during the phases 1 and 2, respectively.

^4)^B, barrow; G, gilt; Sr, sire; PN, plane of nutrition; S, sex.

The ADG during the phase 2 was greater in the H-sires’ progeny vs. the M-sires’ progeny (0.72 vs. 0.66 kg; SE = 0.01 kg; P < 0.01), which was opposite to the result of the phase 1. On the other hand, phase 2 ADG was greater (P < 0.01) in the H and M groups vs. the L group (0.74, 0.71, and 0.61 kg for the H, M, and L groups, respectively; SE = 0.02 kg) as in the phase 1. The ADG for the entire grower phase did not differ between the two progeny groups. Within the M-sires’ progeny, overall ADG was greater in the H vs. M group (P < 0.05) and also in the M vs. L group (0.66, 0.61, and 0.49 kg for the H, M, and L groups, respectively; P < 0.01). However, in the H-sires’ progeny, overall ADG was greater in the H and M group vs. the L group, with no difference between the H and M groups (0.63, 0.62, and 0.54 kg for the H, M, and L groups, respectively).

### Finisher phase

Results for the M-sires’ and H-sires’ progenies during the finisher phase, which included the effects of the grower diet, finisher diet, sex, and their interactions, are shown in Tables 4 and 5, respectively. The effect of the grower × finisher interaction was significant in all variables due to the unbalanced and partially cross-ward transition of the planes of nutrition at the turn of the finisher phase, but no difference was detected between the two sub-groups of animals fed the H- and M-plane grower diets within the group of animals fed the same H-plane (Table 4) or M-plane (Table 5) finisher diet. For these reasons, results for the finisher phase were limited to comparison of growth performance of the animals on the M- or H-plane finisher diet vs. the L-plane finisher unless the effect of sex was significant.

**Table 4:** **Effects of the plane of nutrition on growth of finishing pigs born from the sires with a medium weight gain potential**

	HP Gr^1)^		MP Gr^1)^		LP Gr^1)^			***P***-value				
Item	HP F^1), 2)^		HP F^1), 2)^		LP F^1), 3)^		SEM	Gr	F	S^1)^	Gr × F	F × S
	B^1)^	G^1)^	B	G	B	G						
Initial wt, kg	48.4	52.3	45.7	53.6	43.8	44.2	1.8	<0.01	<0.01	<0.01	<0.01	0.09
Days on feed												
Phase (P) 1	28	29	28	28	35	36						
P 2	41	39	42	38	46	45						
Final wt, kg												
P 1	77.1	78.9	73.4	82.7	71.8	72.1	2.2	<0.01	<0.01	0.03	<0.01	0.15
P 2	113.4	111.2	109.8	113.2	116.7	114.2	2.4	0.26	0.12	0.84	<0.01	0.42
ADG												
P 1	1.01	0.92	0.99	1.04	0.80	0.77	0.04	<0.01	<0.01	0.58	<0.01	0.65
P 2	0.88	0.85	0.86	0.82	0.98	0.93	0.04	<0.01	<0.01	0.15	<0.01	1.00
Overall	0.94	0.88	0.91	0.90	0.90	0.86	0.03	0.56	0.28	0.11	<0.01	0.71
Days to 115 kg	184.3	182.1	190.5	178.5	192.5	196.2	3.3	<0.01	<0.01	0.11	<0.01	0.08
Carcass wt, kg	84.7	85.0	83.4	84.9	88.4	84.9	2.0	0.42	0.21	0.66	<0.01	0.22
Dressing, %	74.6	75.9	75.9	75.0	75.7	74.4	0.8	0.90	0.70	0.61	<0.01	0.22
BFT^4)^, mm	25.1	22.0	25.8	20.5	25.8	26.4	1.2	0.03	0.01	0.01	<0.01	0.03

^1)^HP, high plane; MP, medium plane; LP, low plane; Gr, grower; F, finisher; B, barrow; G, gilt; S, sex. Data are least squares means of 10 animals in each plane of nutrition × sex.

^2)^Provided with the HP and MP finisher diets during the finisher phases 1 and 2, respectively (see Table 1). The average ADFI during the phases 1 and 2 were 2.64 and 3.14 kg, respectively.

^3)^Provided with the LP finisher during both phases 1 and 2 (see Table 1). The average ADFI during the phases 1 and 2 for pigs born from both groups of sires with the medium and high (see Table 5) weight gain potentials were 2.89 and 4.10 kg, respectively.

^4)^Average of backfat thickness measurements between the 11 and 12th ribs and at the last rib adjusted for a 115-kg liveweight.

**Table 5:** **Effects of the plane of nutrition on growth of finishing pigs born from the sires with a high weight gain potential**

	HP Gr^1)^		MP Gr^1)^		LP Gr^1)^			***P***-value				
Item	MP F^1), 2)^		MP F		LP F^1), 3)^		SEM	Gr	F	S^1)^	Gr × F	F × S
	B^1)^	G^1)^	B	G	B	G						
Initial wt, kg	52.8	47.0	53.7	47.0	45.8	44.5	1.9	0.01	<0.01	<0.01	<0.01	0.10
Days on feed												
Phase (P) 1	31	31	30	31	37	37						
P 2	38	38	37	37	38	38						
Final wt, kg												
P 1	82.2	78.3	82.4	76.1	81.8	78.0	2.8	0.95	0.96	0.05	<0.01	0.80
P 2	119.3	111.0	111.4	109.9	121.5	118.3	3.3	0.02	0.01	0.11	<0.01	1.00
ADG												
P 1	0.95	1.01	0.95	0.95	0.97	0.91	0.04	0.60	0.50	0.94	<0.01	0.18
P 2	0.97	0.86	0.81	0.91	1.05	1.06	0.04	<0.01	<0.01	0.87	<0.01	0.79
Overall	0.96	0.93	0.88	0.93	1.01	0.98	0.03	<0.01	<0.01	0.95	<0.01	0.35
Days to 115 kg	178.8	186.8	178.2	179.7	180.7	184.5	4.1	0.58	0.72	0.07	<0.01	0.38
Carcass wt, kg	89.3	81.8	84.8	85.2	87.7	86.9	2.1	0.48	0.23	0.13	<0.01	0.51
Dressing, %	74.8	73.7	74.5	75.4	73.9	73.4	0.8	0.33	0.23	0.78	<0.01	0.76
BFT^4)^, mm	21.7	23.3	23.0	25.9	26.5	26.0	1.1	<0.01	<0.01	0.31	<0.01	0.10

^1)^HP, high plane; MP, medium plane; LP, low plane; Gr, grower; F, finisher; B, barrow; G, gilt; S, sex. Data are least squares means of 10 animals in each plane of nutrition × sex.

^2)^Provided with the MP and LP finisher diets during the finisher phases 1 and 2, respectively (see Table 1). The average ADFI during the phases 1 and 2 were 2.40 kg and 3.96 kg, respectively.

^3)^Provided with the LP finisher during both phases 1 and 2 (see Table 1). The average ADFI during the phases 1 and 2 for the pigs born from both groups of sires with the medium (see Table 4) and high weight gain potentials were 2.89 and 4.10 kg, respectively.

^4)^Average of backfat thickness measurements between the 11 and 12th ribs and at the last rib adjusted for a 115-kg liveweight.

In the progeny of the M-sires (Table 4), ADG during the finisher phase 1 was greater (P < 0.01) on the H-plane finisher diet vs. the L-plane finisher (0.99 vs. 0.78 kg). However, ADG during the finisher phase 2 was greater in the group on the L-plane finisher diet during both phases 1 and 2 (L group) than in the other group on the H- and M-plane finishers during the phases 1 and 2, respectively (H group; 0.96 vs. 0.85 kg). Consequently, ADG during the entire finisher period did not differ between the L and H groups (0.91 vs. 0.88 kg). The ADG was not influenced by sex during either phase or the entire finishing period. The estimated number of days required to reach 115 kg of liveweight in the L group was greater than that in the H group (194.3 vs. 183.9 d), which was largely attributable to the former having 6.6 kg lower BW at the beginning of the finisher phase. However, the number of days required to reach the 115-kg liveweight was not influenced by sex. Carcass weight and dressing percentage did not differ between the dietary groups or sexes. Backfat thickness at slaughter adjusted for the 115-kg liveweight was greater (P < 0.01) in the L group than in the H group (26.1 vs. 23.4 mm; SE = 0.7 mm) as well as in barrows vs. gilts (25.6 vs. 23.8 mm). Furthermore, backfat thickness was greater in barrows than in gilts in the H group (P < 0.01), but in the L group, it did not differ between the two sexes.

In those pigs born from the H-sires, ADG during the phase 1 did not differ between the L group and the M group fed the M- and L-plane finisher diets during the phases 1 and 2, respectively (Table 5). However, during the phase 2 when all pigs were fed the L-plane finisher, the L group exhibited a greater ADG than the M group (1.05 vs. 0.88 kg; P < 0.01). Likewise, ADG during the entire finisher phase was greater in the L group vs. M group (1.00 vs. 0.93 kg; P < 0.01). However, ADG was not influenced by sex during either finisher phase. The estimated number of days required to reach the 115-kg liveweight was not influenced by either sex or plane of nutrition (181.1 vs. 182. 6 for the M and L groups, respectively), even though BW at the beginning of the finisher phase was 5.0 kg less in the L group vs. the M group. Carcass weight and dressing percentage were not influenced by the dietary treatment or sex. Backfat thickness adjusted for the 115-kg liveweight was greater (P < 0.01) in the L group than in the M group (26.3 vs. 23.5 mm; P < 0.01).

Overall, the number of days required to reach the 115-kg liveweight and the liveweight-adjusted backfat thickness of the H group of the M-sires’ progeny on the H plane during the entire growing-finishing period did not differ from those of the M group of the H-sires’ progeny (183. 2 vs. 185.6 d and 23.7 vs. 24.4 mm, respectively) when the raw data of Tables 4 and 5 were pooled and further analyzed. In those pigs on the L plane throughout the growing-finishing period, the number of days required to reach the 115-kg liveweight was less (P < 0.01) in the H-sires’ progeny than in the M-sires’ progeny (182.6 vs. 194.0 d; SE = 2.7 d), whereas the adjusted backfat thickness did not differ between the two groups (26.3 and 26.1 mm in the former and latter, respectively).

## Discussion

Main results of the present study are as follows. The M-sires’ progeny grew faster than the H-sires’ progeny during the early grower phase whereas during the latter grower phase, the H-sires’ progeny grew faster. The M group of the H-sires’ progeny gained weight as well as the H group of the M-sires’ progeny throughout the growing-finishing period. Moreover, the L group of the H-sires’ progeny grew faster than that of the M-sires’ progeny during the entire growing-finishing period. Collectively, these results suggest that the H plane of nutrition is necessary to elicit maximal weight gain in the M-sires’ progeny during the grower phase beginning from early August whereas the M plane is sufficient for the H-sires’ progeny. Moreover, during the early finisher phase, the L plane is probably sufficient for maximal weight gain for the H-sires’ progeny whereas the H or M plane is necessary for the M-sires’ progeny.

Unexpectedly, both L groups of the M- and H-sires’ progenies exhibited greater ADG during the latter finisher phase and greater backfat thickness at slaughter than the H and M groups of the respective progeny groups. It was also evident that feed intake was greater in the L group vs. the M or H group during the latter finisher phase, although only the average intake of each diet was measured due to practical limitations of the commercial farm where the present experiment was performed. These responses of the L group, which are consistent with the typical phenomena of the animals undergoing compensatory growth [20], suggest that the accelerated growth of the L group during the latter finisher phase resulted from compensatory growth. In this regard, the relatively long lag period between growth retardation and compensatory growth in the L group seems to be related to the long period of inadequate nutrition during the early developmental stage when the growth retardation occurred [20, 21]. It was not clear, however, whether or not the apparently high ADG of the M and H groups during the early finisher phase (0.95 to 1.00 kg) also resulted from compensatory growth. Further, the daily high ambient temperature during this period frequently exceeded 30°C (data not shown), which is far above 26 to 28°C of the threshold temperature causing the heat stress in growing-finishing pigs [22, 23]. Accordingly, it also remains unknown how much of the observed growth rates of the whole animals were influenced by a presumptive heat stress during the grower phase. Further studies are therefore warranted to determine the effects of the heat stress and plane of nutrition during the hot summer as well as their interactions on subsequent growth on varying planes of nutrition during the fall.

According to the grading standard for pig carcasses, the upper and lower limits of carcass weight for the 1^+^ grade are 83.5 and 93.5 kg, respectively in Korea [24], which are equal to 109.9 and 123.0 kg by the liveweight, respectively, at a dressing percentage of 76%. In addition, backfat thickness should fall within a range between 17.5 and 24.5 mm to be eligible for the 1^+^ grade by Korean standards, which means that the optimum backfat thickness of market pigs is 21.5 mm if a standard deviation of 3 mm [25] is deducted from the upper limit of the 1^+^ grade as a safety margin. As such, the average backfat thickness of both M- and H-sires’ progenies at the market weight, which was optimal on the M or H plane (23.5 mm), but not on the L plane (over 26 mm), under the previous carcass grading standard (26.5 mm maximum to be eligible for the 1^+^A grade; [16]), is now too high by the current standard. Evidently, sires leaner than those used in the present study are now needed not only to meet the current grading standard calling for leaner carcasses but also to improve feed efficiency.

## Conclusions

The present results suggest that the H-to-H or H-to-M planes and M-to-M or M-to-L planes are optimal for maximal growth of the M- and H-sires’ progenies, respectively.
